# Influence of the work environment of nurses on the 30-day mortality of patients hospitalized in Polish hospitals. cross-sectional studies

**DOI:** 10.1186/s12912-024-01762-x

**Published:** 2024-02-15

**Authors:** Iwona Malinowska-Lipień, Dariusz Put, Michał Maluchnik, Teresa Gabryś, Maria Kózka, Krzysztof Gajda, Agnieszka Gniadek, Tomasz Brzostek, Allison Squires

**Affiliations:** 1 Institute of Nursing and Midwifery, Faculty of Health Sciences, Jagiellonian University– Medical College, Kopernika Str. 25, 31-501 Krakow, Poland; 2 Department of Computational Systems, Krakow University of Economics, Krakow, Poland; 3 Department of Adult Neurology, Medical University of Gdansk and University Clinical Center, Gdansk, Poland; 4 Ministry of Health, Warsaw, Poland; 5 Institute of Public Health, Faculty of Health Sciences, Jagiellonian University– Medical College, Krakow, Poland; 6 Rory Meyers College of Nursing, New York University, New York, USA

**Keywords:** Work environment, Nurses, Patients, 30-day mortality

## Abstract

**Background:**

An optimal work environment for nurses is characterized primarily by appropriate staffing, good team relations, and support from the management staff. These factors are consistently associated with a positive assessment of patient safety by a hospital’s employees and a reduction in hospital mortality rates.

**Aim:**

To understand the relationships between the work environment as perceived by nurses on the 30-day mortality of patients treated in Polish hospitals.

**Background:**

An optimal work environment for nurses is characterized primarily by appropriate staffing, good team relations, and support from the management staff. These factors are consistently associated with a positive assessment of patient safety by a hospital’s employees and a reduction in hospital mortality rates.

**Material and methods:**

The analysis used discharge data from 108,284 patients hospitalized in internal medicine and surgery departments in 21 hospitals (with 24/7 operations) in Poland. Administrative data included coded data to estimate 30-day mortality. A Nurses’ satisfaction questionnaire, including the PES-NWI scale and the SAQ questionnaire, was used to assess the work environment of nurses (*n* = 1,929). Correlations between variables were assessed using the Pearson coefficient. The analysis used a Poisson regression model, which belongs to the class of generalized linear models.

**Results:**

A lower 30-day mortality rate amongst patients was found among those treated in hospitals where the personnel feel that they may question the decisions or actions of their superiors regarding the care provided (*r* = − 0.50); nurses are informed about changes introduced on the basis of reports about negligence and mistakes (*r* = − 0.50); the ward nurse is a good manager (*r* = − 0.41); nurses receive timely information from the head of the department that may have an impact on their work (*r* = − 0.41).

**Conclusions:**

Factors related to care during hospital stay such as the organization of care at the ward level, analysis of care errors, the number of staff providing direct patient care, informing nurses about mistakes without punishment, and the possibility of nurses challenging the decisions or actions of superiors, which concerns care providing, affect the 30-day mortality of patients after the end of hospitalization in Polish hospitals.

## Introduction

The work environment for nurses consists of organizational features that affect the performance of their work. To improve working conditions for nurses, research about the environment where they work is essential as it generates the necessary data from which management and leadership can begin to tackle the sources of poor working conditions, occupationally-related burnout, and issues with employee retention. By improving the work environment, leadership and management have the potential to positively influence the results of treatments and the health condition of hospitalized patients [1–4].

The work environment, therefore, is a modifiable factor of where nurses work. Autonomy, good relationships, and teamwork are considered to be beneficial factors in the work environment of nurses, which bring better results both for the institution that provides care, as well as for nurses and patients [5]. A well-functioning work environment is associated with a higher assessment of the quality of nursing care, a higher level of job satisfaction [5] and a lower level of occupational burnout and staff turnover [4] Moreover, studies show that improving the work environment is associated with a positive assessment of patient safety [6], an increase in the level of patient satisfaction [5], and a reduction in the mortality of hospitalized patients [2, 7]. The largest group of medical workers are nurses who are responsible for direct patient care and provide most health services. There is growing evidence that the incidence of adverse events such as patient falls, healthcare-associated infections, medication errors, and in-hospital mortality are associated with insufficient nursing staff [8, 9].

International studies have shown that “Magnet Hospitals” attract and retain specialists because they provide high-quality care, patient safety, cooperation in an interdisciplinary team, positive communication, professional development opportunities, and a better working environment than other hospitals [10–11]. On the other hand, nurses who work in hospitals with more traits that do not meet the above criteria are less likely to be satisfied with their work [12], are more likely to report their intention to quit their jobs [13], and are more prone to burnout [14].

In addition, it has been shown that inadequate nursing staffing and heavy workloads result in reduced quality of nursing care and increased patient mortality rates [15]. The results of studies conducted in nine European countries showed that each patient additionally added to nursing care was associated with a 7% higher risk of patient death within 30 days from admission [7]. Numerous studies have shown that more qualified nurses working in hospital departments are associated with better treatment outcomes, including fewer nosocomial infections, shorter hospital stays, fewer readmissions, higher patient satisfaction, and lower occupational burnout among nurses [6, 11, 16–18].

The shortage of nurses is a serious problem in the healthcare system all over the world. In Poland, multiple studies have documented the negative impact of the nursing shortage on patient care [1, 7, 19]. Nurse shortages as well as other work environment constraints also have a negative impact on the physicians’ practice, as they work with fewer human and environmental resources [1, 19–20].

The effectiveness of health care depends not only on the knowledge and skills of medical staff but also on the working conditions in which they work. Understanding how nurses’ work environments impact the quality of care can lead to improvements in the healthcare system.

The study can provide information on the efficiency of resource use in medical facilities. Identifying problem areas in nurses’ work environments can help optimize resource allocation and improve the overall quality of care.

### Aim of the research

To understand the relationships between the work environment as perceived by nurses on the 30-day mortality of patients treated in Polish hospitals.

## Material and method

### Design

The cross-sectional study was conducted from December 2018 to April 2019 in a sample of 21 hospitals on 24-hour constant duty representative for Poland. Hospitals were selected according to the region of the country, population density, and different levels of reference in accordance with the requirements of the RN4CAST study protocol carried out in 2009–2011 [19]. These were 21 hospitals out of 30 that participated in the RN4Cast project. At least one of the hospitals was located in one of the 16 voivodeships into which Poland is divided.

The survey was sent to 2,541 nurses. In the process of analysing the collected data, observations with no answers to most of the questions asked were excluded. Ultimately, 1,929 respondents were used for the final analysis.

At the hospital, a designated hospital coordinator was assigned to facilitate communication with the research team. Within clinical departments, the distribution of questionnaires occurred during staff meetings conducted by the hospital project coordinator. Participants were allotted a 4-week timeframe to complete the questionnaire. Completed surveys were deposited into sealed boxes equipped with a collection slot. Following the designated period, the coordinator retrieved the boxes, secured them, and subsequently delivered them to the research team. Participants were explicitly informed that their involvement was voluntary and anonymous. The inclusion criterion was Polish nationality and working as a nurse in an internal medicine or surgical ward. The exclusion criterion was no answers to at least 40% of the survey questions.

### Sample

- Data on 108,284 patients hospitalized in 21 hospitals in Poland in internal medicine and surgical departments.
- Data collected from 1,929 nurses employed in the same hospitals from which the patient data were obtained.

### Measures

The survey was conducted among nurses of twenty-one hospitals in Poland working in internal medicine and surgery departments. To assess the work environment of nurses (*n* = 1,929 nurses), the Nurses Satisfaction Questionnaire, including the PES-NWI (The Practice Environment Scale of the Nursing Work Index) and the SAQ (The Safety Attitudes Questionnaire), were used.

The nurses’ satisfaction questionnaire included three parts. It incorporated the PES-NWI Work Environment Scale, which featured 32 statements covering five aspects of the work environment. These aspects included three assessing the hospital’s adequacy of human and material resources, cooperation in the nurse-physician therapeutic team, and support received by nurses from the managerial staff. Additionally, there were two aspects focusing on the participation of nurses in hospital management and support for the implementation of nursing care quality standards. This part also included questions about the overall assessment of working conditions and satisfaction with work in the hospital in terms of flexibility in working time planning, independence at work, professional status, satisfaction with the level of remuneration, promotion opportunities, education, obtaining annual and training leave, and using sick leave. The second part of the questionnaire concerned the assessment of patient safety and the quality of nursing care. The third part contained information on the last on-call duty performed by the nurses, which was used to assess the workload, the participation of non-qualified personnel in the care, and information on the activities that the nurses did not manage to perform during their on-call duty due to a lack of time. The reliability (i.e., Cronbach alpha coefficients) of the PES-NWI subscales vary from 0.71 to 0.84 [19]. Cronbach’s alpha coefficients of the SAQ subscales for the study group ranged from 0.84 to 0.90.

The SAQ questionnaire (in Polish adaptation [21]) consisted of 41 items divided into two parts. The first contained 36 questions divided into 6 subscales, the second contained 5 questions concerning the sociodemographic data of the participants. The first part was 1/Teamwork climate (TC) (questions 1 to 6)– assesses the perception of the quality of cooperation between employees; 2/Safety climate (SC) (questions 7 to 13)– assesses the perception of employees’ organizational commitment to safety; 3/Job satisfaction (JS) (questions 15 to 19)– assesses subjective feelings related to professional experience; 4/Stress recognition (SR) (questions 20 to 23)– assesses the impact of stressors on performance at work; 5/Perception of management (PM) (questions 24 to 28)– assessed at the department and hospital level; and 6/Work conditions (WC) (questions 29 to 32)– perception of the quality of community and logistics support in the workplace (e.g., equipment, devices, and professionals), as well as five questions not included in any of the subscales, i.e., question 14 relating to the assessment of the manager in terms of ensuring safety and questions from 33 to 36 concerning the assessment of the occurrence of conflicts and quality cooperation between members of the interdisciplinary team, i.e., nurses, physicians, or pharmacists. The reliability (i.e., Cronbach alpha coefficients) of the SAQ subscales vary from 0.56 to 0.95 [21]. Cronbach’s alpha coefficients of the SAQ subscales for the study group ranged from 0.48 to 0.85.

The nurses’ working environment, including determinants of attitudes towards safety, was analysed from the hospital’s perspective, while 30-day mortality was analysed from the patient’s perspective.

The statistical analysis included data from reports submitted by hospitals to the payer and was an anonymised fragment of the report submitted to the National Health Fund. Selected data of patients’ discharge were anonymous and compliant with the Personal Data Protection Act and the principles of ethical social research guaranteeing anonymity. The patient’s personal data (name and surname, personal identification number) was deleted by coordinators at the hospital level [22]. The information necessary for the analysis obtained from the report to the National Health Fund (NHF) included age, gender, discharge mode, and the department where the patient was hospitalized. In line with the assumption, the studies did not concern paediatric, psychiatric, gynaecological, and obstetric departments. The data for the analysis included patients who were at least 18 years of age on admission. The dependent variable was the death rate, defined as the ratio of the number of deaths to the number of admissions, calculated on the basis of information about the patient’s discharge. The primary endpoint was patient mortality 30 days after discharge from hospital.

The independent (explanatory) variables were obtained from the Nurses Satisfaction Questionnaire, including the PES-NWI (The Practice Environment Scale of the Nursing Work Index) as well as the Safety Attitudes Questionnaire (SAQ), which were conducted among employed nurses who provided direct patient care.

The research was conducted in accordance with the principles of the Helsinki Declaration, after obtaining the consent of the Bioethics Committee of the Jagiellonian University no. 1072.6120.111.2018.

### Data analysis

The statistical analysis of the data was carried out with the use of *Excel*, the *NumPy* and *SciPy Python* language libraries, and also the *confintr* and *glmnet* packages of *R* language.

Qualitative variables were characterised presenting the number of cases (n) and percentage (%).

In the survey, correlation coefficients of individual explanatory variables obtained from both the nurses’ professional satisfaction survey and the questionnaire conducted among nurses with the mortality rate as the dependent variable were calculated first. Then, an attempt was made to find a linear regression model for various potentially justified combinations of explanatory variables of the SAQ questionnaire. Because the dependent variable, which represents the number of deaths within 30 days from discharge, is a count data type, additional analysis was conducted using the Poisson regression to model the relationship between it and selected regressors of all 137 variables from the nurses’ satisfaction questionnaire. The analyses enabled finding significant factors potentially influencing the number of deaths within 30 days from discharge.

Correlations between variables were assessed using the Pearson coefficient. The analysis used a Poisson regression model, which belongs to the class of generalized linear models. Generalized linear models enable testing the impact of categorical and continuous predictors on a dependent variable whose distribution belongs to the family of exponential distributions, and independent variables may influence the dependent variable in a non-linear manner. Estimation and verification of all models were performed using the system. The construction of the model used in the study began with the estimation of several Poisson regression models using the classical method. Then, using basic measures of goodness of fit to the observed data and examining the correlation between variables, the best model was selected.

For all analyses, the maximum allowable type I error was assumed to be *α* = 0.05, while *p* < 0.05 was considered statistically significant.

## Results

Discharge data concerning 108,284 patients hospitalized in internal medicine and surgery departments in 21 hospitals in Poland were used for the analysis; the number of patients and the number of patients who died varied from hospital to hospital. The total number of deaths in the analysed group of hospitals was 3,589. The percentage of patients who died per hospital within 30 days of discharge varied between 21 participating hospitals (Min = 1.90%, Max = 5.46%), on average it was 3.42%; Table 1.

**Table 1:** The number of patients admitted to internal medicine and surgery departments and the number of deaths up to 30 days after discharge in individual hospitals included in the study

Hospital code	Patient number	Number of patients who died up to 30 days after discharge	Percentage of patients who died up to 30 days after discharge	Confidence Interval Lower boundary (2.5%)	Confidence Interval Upper boundary (97.5%)
11	4149	115 W: 61 M: 54	2.77%	2,29%	3,32%
12	5564	160 W: 84 M: 76	2.88%	2,45%	3,35%
13	5996	196 W: 90 M: 106	3.27%	2,83%	3,75%
15	3318	63 W: 33 M: 30	1.90%	1,46%	2,42%
16	4588	161 W: 86 M: 75	3.51%	3,00%	4,08%
17	3380	176 W: 95 M: 81	5.21%	4,48%	6,01%
18	8308	199 W: 111 M: 88	2.40%	2,08%	2,75%
19	4238	138 W: 68 M: 70	3.26%	2,74%	3,84%
20	4417	241 W: 120 M: 121	5.46%	4,80%	6,17%
21	11,075	269 W: 133 M: 136	2.43%	2,15%	2,73%
22	2484	68 W: 39 M: 29	2.74%	2,13%	3,46%
24	3519	121 W: 59 M: 62	3.44%	2,86%	4,09%
27	2908	101 W: 59 M: 42	3.47%	2,84%	4,20%
28	3382	95 W: 49 M: 46	2.81%	2,28%	3,42%
29	10,310	476 W: 251 M: 225	4.62%	4,22%	5,04%
30	8555	431 W: 212 M: 219	5.04%	4,58%	5,52%
32	2540	49 W: 22 M: 27	1.93%	1,43%	2,54%
35	4880	146 W: 77 M: 69	2.99%	2,53%	3,51%
38	4697	210 W: 99 M: 111	4.47%	3,90%	5,10%
39	3732	159 W: 76 M: 83	4.26%	3,64%	4,96%
40	6244	130 W: 69 M: 61	2.08%	1,74%	2,47%
Total	108 284	3 704 W: 1893 M: 1811	3.42%	2,29%	3,32%

*W- woman; M- man

The highest percentage of deceased patients from internal medicine and surgery wards was in the range of 80 to 89 years (32.13%). The death rate increased significantly for the group over 60 years of age and was highest for patients over 80 years of age. The results remain in line with expectations. Table 2.

**Table 2:** Age of patients who died up to 30 days after discharge in individual hospitals included in the study

Hospital code		Age ranges of patients who died up to 30 days after discharge							
		18–29	30–39	40–49	50–59	60–69	70–79	80–89	> 90
11		0	3	1	10	26	25	31	19
12		1	4	4	17	34	31	51	18
13		0	3	5	8	38	57	57	28
15		0	0	1	2	11	13	29	7
16		0	1	2	13	48	29	51	17
17		0	1	3	14	35	48	57	18
18		3	2	6	18	52	43	51	24
19		1	1	2	7	18	29	57	23
20		0	0	4	20	46	54	82	35
21		0	7	12	15	67	53	87	28
22		0	0	4	6	11	17	27	3
24		0	1	3	9	23	29	43	13
27		0	0	0	1	18	17	48	17
28		0	3	2	7	19	19	31	14
29		2	2	7	19	96	123	147	80
30		1	2	11	24	90	98	145	60
32		1	0	2	12	15	8	9	2
35		0	4	2	13	35	30	42	20
38		0	1	7	15	55	45	69	18
39		1	1	3	15	32	43	46	18
40		1	1	2	13	34	35	30	14
Total		11	37	83	258	803	846	1190	476
		0.30%	1.00%	2.24%	6.97%	21.68%	22.84%	32.13%	12.85%

### Nurses working environment

There were moderate correlation coefficients (0.40 to 0.60) between the 30-day mortality rate and individual explanatory variables, obtained from the nurses’ professional satisfaction survey (PES-NWI) (Table 3). A lower 30-day mortality was found among patients hospitalized in hospitals where:

- the ward nurse was assessed as a good manager (*r* = − 0.41),
- nurses were informed about changes introduced on the basis of reports about negligence and mistakes (*r* = − 0.50),
- staff felt that they might question their supervisor’s decisions or actions regarding their care (*r* = − 0.50),
- the number of other medical personnel providing direct care to patients was greater (*r* = − 0.48).

Higher 30-day mortality was found among patients hospitalized in hospitals where the employed staff felt that they were severely punished for their mistakes without forgiveness (*r* = 0.45) and when services were organized to ensure continuity of care by the same person (i.e., the same nurse looked after the patient during long term nursing duty), (*r* = 0.53).

The following parameters were demonstrated to have a weak correlation coefficient (from 0.2 to 0.4) with lower 30-day mortality rates among hospitalized patients:

- a job well done was praised and appreciated (*r* = − 0.31),
- the ward nurse supported the nursing staff even in a conflict with the physicians (*r* = − 0.33),
- nurses were satisfied with the flexible work schedule (*r* = − 0.31).

However, the 30-day mortality rate was higher (with weak correlation coefficients from 0.20 to 0.40) when:

- staff felt exhausted at the end of the working day (*r* = 0.34),
- staff felt tired when they got up for work in the morning (*r* = 0.34),
- staff felt they were working too hard (*r* = 0.33),
- nurses experienced physical abuse by patients and/or their families (*r* = 0.36),
- after admitting the patient to the ward, they developed pressure ulcers (*r* = 0.33),
- nurses were involved in the preparation of discharge and transport (*r* = 0.30).

In the case of the SAQ questionnaire, the largest negative correlation was equal to.

*r* = -0.41 (question: I receive timely information from the head of the department that may have an impact on my work). The value of the correlation coefficient indicates a weak relationship with mortality but suggests that timely information about an employee and their job may contribute to reducing mortality. In three cases, the correlation coefficient with the dependent variable was greater than 0.30. The largest positive correlation shown was:

- In a situation of tension or hostility, the risk of making a mistake is greater (*r* = 0.38),
- Fatigue has a negative impact on my work in emergencies (e.g., resuscitation, epilepsy), (*r* = 0.36)
- Management is not knowingly putting patient safety at risk (*r* = 0.36).

**Table 3:** Selected correlation coefficients of individual explanatory variables obtained from the questionnaire conducted among nurses with the mortality rate

Independent variable	Negative correlation with mortality rate
staff felt that they might question their supervisor’s decisions or actions regarding their care	-0.50
nurses are informed about changes introduced on the basis of reports about their negligence and mistakes	-0.50
the number of other medical personnel providing direct care to patients was greater in relation to the number of nurses	-0.48
the ward nurse was a good manager	-0.41
nurses receive timely information from the head of the department that may have an impact on their work	-0.41
the ward nurse supported the nursing staff even in a conflict with the physicians	-0.33
a job well done was praised and appreciated	-0.31
nurses were satisfied with the flexible work schedule	-0.31
	**Positive correlation with mortality rate**
services were organized to ensure continuity of care (i.e., the same nurse looked after the patient for a long time on call)	0.53
the employed staff felt that they were severely punished	0.45
in a situation of tension or hostility, the risk of making a mistake is greater	0.38
fatigue has a negative impact on nurse’s work in emergencies	0.36
management is not knowingly putting patient safety at risk	0.36
nurses experienced physical abuse by patients and/or their families	0.36
staff felt exhausted at the end of the working day	0.34
staff felt tired when they got up for work in the morning	0.34
staff felt they were working too hard	0.33
after admitting the patient to the ward, they developed pressure ulcers	0.33
nurses were involved in the preparation of discharge and transport	0.30

On the other hand, an affirmative answer to question 28: I receive timely information from the head of the department that may have an impact on my work, was demonstrated to be correlated with lower 30-day mortality among hospitalized patients (*r* = − 0.41).

The aggregation of data from the SAQ questionaries, filled in by the nurses, had no additional significant effect on the 30-day mortality of hospitalized patients. However, the recognition of work stress (SR) (*r* = 0.28) was weakly correlated with higher 30-day mortality, while a positive perception of the management method (PM) (*r* = -0.27) correlated with lower 30-day mortality among hospitalized patients.

### Results of the regression analysis

In the case of the nurse satisfaction questionnaire, five variables were selected for the construction of the econometric model for which the module of the correlation coefficient with mortality was at least 0.40 and for which the substantive relationship with patient mortality would be justified (Table 4).

**Table 4:** Variables selected for the construction of the econometric model (Nurses Satisfaction Questionnaire)

Explanatory variable	Correlation coefficient with mortality
x1	-0.41
X2	0.53
X3	0.45
X4	-0.50
X5	-0.48

x1- The ward nurse is a good manager and organizer; x2- The care is organized in such a way as to ensure its continuation (i.e., the same nurse looks after the patient for some time); x3- Staff feels that they are severely punished for their mistakes without forgiveness; x4- Staff feels they can challenge the decisions or actions of their supervisors; x5- Total number of other staff providing direct care to patients in your ward during your last on-call service

Before starting to build the model, the correlation coefficients between the potential explanatory variables were calculated (Table 5).

**Table 5:** Coefficients of correlation between explanatory variables with the most significant impact on mortality (according to the Nurses Satisfaction Questionnaire)

	X1	X2	X3	X4	X5
X1	1	0.20386	-0.26576	**0.543772**	0.184521
X2		1	0.082524	0.170391	-0.31188
X3			1	**-0.59226**	**-0.62947**
X4				1	0.483423
X5					1

x1- The ward nurse is a good manager and organizer; x2- The care is organized in such a way as to ensure its continuation (i.e., the same nurse looks after the patient for some time); x3- Staff feels that they are severely punished for their mistakes without forgiveness; x4- Staff feels they can challenge the decisions or actions of their supervisors; x5- Total number of other staff providing direct care to patients in your ward during your last on-call service

In order to limit the interaction between variables in the constructed econometric model, the explanatory variables for which the correlation coefficient modulus was greater than 0.5 were not taken into account simultaneously. Then, all possible theoretically justified econometric models were built. The highest coefficient of determination and a maximum of one statistically insignificant parameter were obtained for four models with three explanatory variables. The best fit between these models was: Y = 0.099–0.014*X1 + 0.022*X2–0.03*X4 with a determination coefficient of 0.71, although, in this model, the influence of the X1 variable on the explained variable turned out to be statistically insignificant.

In the case of models with up to two explanatory variables, only those in which all parameters turned out to be statistically significant were taken into account. The best fit of those models was: Y = 0.086 + 0.021*X2–0.04*X4 with a coefficient of determination of 0.65; and Y = 0.060 − 0.025*X1 + 0.021*X2 with a coefficient of determination of 0.57. The list of models and parameters is presented in Table 6.

**Table 6:** Econometric models with the best parameters (according to the Nurses Satisfaction Questionnaire)

	A0	A1	A2	A3	A4	A5	R2	**Parameter significance**	Model error
**Best models with three explanatory variables**									
	0.016	-0.021	0.020	0.013			0.64	a3 insignificant	0.007
	0.099	-0.014	0.022		-0.03		0.71	a1 insignificant	0.006
	0.073		0.021	0.003	-0.039		0.65	a3 insignificant	0.007
	0.087		0.022		-0.042	0.0006	0.64	a5 insignificant	0.007
**Best models with two explanatory variables**									
	0.060	-0.025	0.021				0.57	significant	0.007
	0.086		0.021		-0.04		0.65	significant	0.007

A0– free word

A1– A5– coefficients for the variables X1–X5

A1– x1- The ward nurse is a good manager and organizer; x2- The care is organized in such a way as to ensure its continuation (i.e., the same nurse looks after the patient for some time); x3- Staff feels that they are severely punished for their mistakes without forgiveness; x4- Staff feels they can challenge the decisions or actions of their supervisors; x5- Total number of other staff providing direct care to patients in your ward during your last on-call service

Two models with two explanatory variables turned out to be statistically significant and were characterized by a determination coefficient greater than 0.5. In all models, the variable X2 was present with an almost identical value of the parameter A. This means that if the hospital provides continuity of care, the mortality increases (an increase in the survey results by 1 increases the mortality by 0.02). None of the models in which four or all five explanatory variables were taken into account had any properties that would prove useful for the description of the studied phenomenon. None of the models with one explained variable had a sufficiently high coefficient of determination.

### The results of Poisson regression and LASSO selection method of model regressors based on the nurses’ satisfaction questionnaire

Because the dependent variable, which represents the number of deaths within 30 days from discharge, is a count data variable, additional analysis was conducted using the Poisson regression to model the relationship between it and selected regressors. This dependent variable takes non-negative integer values, so count data regression models (such as Poisson regression) are suitable to model its conditional distribution. As potential model regressors, all 137 variables from the nurses’ satisfaction questionnaire were taken. The sample contains only 21 observations, so taking into consideration the number of potential regressors which surpass the number of observations, only estimators including a penalty term such as LASSO or Elasticnet can be used to specify and estimate parameters of the Poisson regression model (with logarithm link function, which relates dependent variable conditional expectations with linear combinations of Poisson model regressors).

**Fig. 1:**
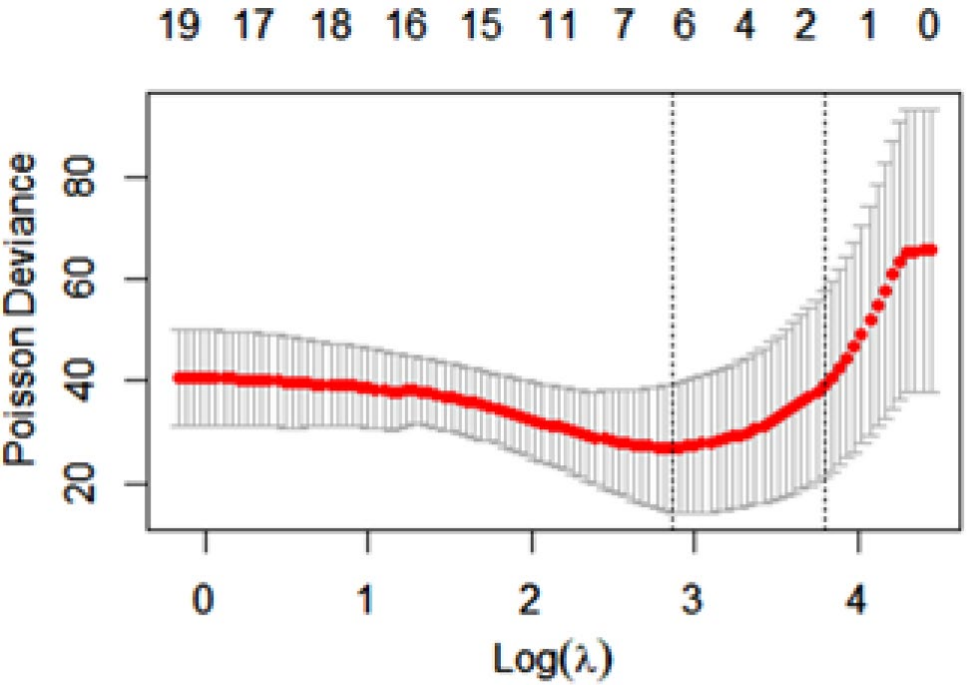
Results of the 3-fold cross-validation LASSO procedure

First, potential regressors were standardized (by subtracting the mean of every value in a feature and dividing the result by standard deviation). To specify the Poisson regression model (i.e., to identify regressors), *k*-fold cross-validation with *k = 3* was used (in each repetition of the cross-validation procedure, 2/3 of sample observations were randomly taken as learning subsamples, and the remaining 1/3 were treated as validation subsamples), in which the Poisson deviance was calculated for validation subsamples as a model fit assessment measure for 100 different values of λ hyperparameter for LASSO estimator criterion for Poisson regression model. To conduct these analyses, the *glmnet* package of *R* language was used. The results of the 3-fold cross-validation LASSO procedure are depicted in Fig. 1. The horizontal axis contains a logarithm of λ hyperparameter for the LASSO estimator and the vertical axis represents values of Poisson deviance.

As the model with the lowest average value of Poisson deviance on validation subsamples, the cross-validation procedure points out a model with six nonzero parameter regressors (including the number of patients offset variable):

- numb_pat– number of patients;
- (a_1_10)– the ward nurse is a good manager;
- (a_9_3)– nurses feel tired when they get up in the morning and another day of work awaits them;
- (b_7_7_1)– nurses are physically insulted by patients and/or their families;
- (c_11_8)– while on duty, nurses often pick up supplies or equipment;
- (c_12_1)– during the last tour of duty, due to lack of time, not all activities according to the rules of patient monitoring were performed.

For comparison, in Table 7, we present the simplest model (i.e., the model with the lowest number of non-zero parameters, which consists here of two parameters: *numb_pat* and *c_12_1*), having Poisson deviance for the validation subsamples, which was not larger by more than 1 standard error for the Poisson deviance mean estimator, calculated over validation subsamples, from the lowest one (here with six nonzero parameters).

**Table 7:** The simplest model data of Poisson analysis

Measure: Poisson Deviance					
	Lambda	Index	Measure	SE	Nonzero
min	17.50	35	26.93	12.53	6
1se	44.38	15	39.29	18.15	2

Lambda - A value of LASSO criterion hyperparameter λ; Index - A number in the sequence of considered λ hyperparameter values; Measure - A mean estimator value of Poisson deviance on validation subsets; SE - A standard error for Poisson deviance mean estimator on validation subsets; Nonzero - A number of potential regressor variables with nonzero values of LASSO parameter estimator.

In Table 8, the LASSO estimator values (calculated on the full sample) for the Poisson regression model with six nonzero parameters selected by a cross-validation procedure are presented.

**Table 8:** LASSO estimator values for the Poisson regression model with nonzero parameters

(Intercept)	4.7888
number of patients	0.0001
(no 10) the ward nurse is a good manager	-0.0478
(no 3) nurses feel tired when they get up in the morning and another day of work awaits them	0.0937
(no 7) nurses are physically insulted by patients and/or their families	0.1029
(no 8) while on duty, nurses often pick up supplies or equipment	-0.2823
(no 12.1) during the last tour of duty, due to lack of time, not all activities according to the rules of patient monitoring were performed	-1.7663

The conditional expected value for the Poisson distribution of the 30-day patient mortality rate is influenced by three regressors with positive impacts and three regressors with negative impacts. A positive impact means that, on average, higher values of specific regressor (with a positive parameter estimate), correspond to higher patient mortality rates, taking everything else as equal. Three of these regressors have an explainable impact on the 30-day patient mortality rate. The mortality decreases if the ward nurse is a good manager, whereas the mortality increases if nurses feel tired when they get up in the morning and another day of work awaits them and if nurses are physically insulted by patients and/or their families.

In Table 8, the LASSO estimator standard errors, *p*-values, and confidence intervals are not presented because, for the LASSO estimator, there is no consensus on a statistically valid method that accounts for the adaptive nature of the estimation (for discussion, see for example 42, 43).

In Table 9, the Poisson regression model diagnostics (Poisson deviance, mean-squared error, mean absolute error) for a LASSO cross-validation specified model with six nonzero parameter regressors are depicted.

**Table 9:** Poisson regression model diagnostics for a LASSO cross-validation specified model with six nonzero parameters regressors

$deviance 7.280467 [1] “Poisson Deviance”
$mse 1179.58 [1] “Mean-Squared Error”
$mae 26.61972 [1] “Mean Absolute Error”

## Discussion

The results of this study showed that the 30-day patient mortality rate is both related to and decreases with the higher total number of nursing and other personnel providing direct care to patients during their hospital stay. These results coincide with the observations of Griffiths et al., which showed that a low employment rate, especially among nurses, adversely affects the functioning of the healthcare system, and thus the health safety of patients [23].

For example, reducing 30-day patient mortality in Polish hospitals is complicated because of staffing shortages among nurses. Poland has the lowest employment rates for nurses per 1,000 inhabitants [24] among the European Union countries. This means that staff shortages (both current and forecast) affect the Polish healthcare system more compared to other countries, and their consequences are more tangible. Currently, 72% of Polish hospitals lack nursing personnel, and 68% of hospitals lack medical personnel. Nurses are among the most difficult to recruit, while employing other medical staff: physiotherapists, midwives, and paramedics, causes much fewer problems [25].

The risk of a continuous decline in the ratio of employed nurses to the number of patients in Poland is worrying. According to the data of the Supreme Council of Nurses and Midwives (NRPiP), the average age of nurses in Poland is currently 53.2 years [26]. Moreover, as many as 63,120 nurses (out of 232,387 actively working nurses) still work in the profession being at retirement age (over 60) or actually receiving a pension [26]. In 2021, 8,755 nurses acquired pension rights, while 5,693 graduates of nursing studies entered the profession, a difference of -3,062 that proves that there is no generational replacement in the nursing profession in Poland [26] and the problem of staff shortages is growing.

We are aware that the strength of the evidence in the current results supporting the relationship between nursing staffing and patient 30-day mortality may be undermined because the research is cross-sectional and uses hospital-level administrative data that does not accurately quantify the number of staff allocated to a given unit and does not take into account the differences in the health of patients. Nevertheless, there are a number of studies conducted in this field that have shown similar conclusions regarding the impact of staffing on patient mortality [4, 8, 23, 27]. Moreover, studies by Griffiths et al. [28] showed that an insufficient number of nurses leads to rationing of time for care, which has a significant impact on the failure to perform certain activities in front of the patient [28]. Often, these are not only direct nursing activities but also a lack of time to provide other medical staff, patients, or family with information related to the care, which translates into patient treatment results, and potentially also to a greater incidence of death, both in hospital and after hospitalization [29]. The present studies have confirmed this fact by showing a positive correlation between patient mortality and the occurrence of communication disorders leading to delays in the provision of care.

The present studies showed a positive correlation between the patient mortality rate and the occurrence of tension, fatigue, exhaustion, and stress among nurses. Two other factors concerning nurses’ well-being and working conditions also have a positive impact on mortality; if nurses feel tired when they get up in the morning and another day of work awaits them and if they are physically insulted by patients and/or their families. Occupational stress is an interaction between the situation in the workplace and the person working there. This situation leads to changes in the mental and physiological state of the employee and affects her/his professional functioning [30]. Any short-term stress– eustress, has a positive effect on the body of the individual, mobilizes them and stimulates action, while chronic stress negatively affects health [31–32]. On the other hand, prolonged negative stress at work may cause resignation from work, conflicts with co-workers, dissatisfaction with work, reduced job satisfaction, limitation of correct and timely decision-making, work fatigue, reduced work efficiency, and reduced quality of nursing care [31]. The results of studies by Parveen et al. showed that occupational stress has a direct or indirect impact on the quality of medical services provided [32].

The present research indicates that the work environment during hospitalization is associated with an increased risk of 30-day mortality following discharge. The reduction in mortality rates was notably linked to effective management practices, particularly when ward nurses were positively evaluated by the staff as adept managers. Additionally, hospitals exhibiting lower mortality rates were characterized by a positive reception of management by the staff. This trend was further observed in settings where nurses were well-informed about changes being implemented based on reports of negligence and mistakes. Moreover, a culture fostering staff empowerment to challenge the decisions or actions of their superiors in matters related to patient care was associated with lower mortality rates. Conversely, however, the death rate was higher when staff felt they were being severely punished for their mistakes. These results are consistent with the results of research by Huang et al., who showed that the highest quality of care provided by nurses depends, among other things, on nurses’ participation in ward decision-making, the model of nursing care, skills, support and leadership of management, and quality of relations between nurses [33]. The present results additionally correspond to the results of Rizkianti & Haryani, who showed that incorrect caring behaviour by staff is influenced not only by individual factors but also by work planning and leadership style [34].

The authors of this study sought to find a positive correlation between 30-day patients’ mortality and the fact that the hospital provides greater follow-up care (an increase in survey scores by 1 resulted in an increase in mortality by 0.02). There can be many serious reasons for this, including staff fatigue; many Polish nurses work several jobs in different places. Research carried out in one of the regions of Poland showed that 44.0% of the respondents worked in two workplaces, including 93.5% as a nurse, completing additional jobs for about 160 h a month (39.0%), and a total work time of over 320 h a month. The decisive reason for taking up additional employment (93.3% of respondents) was mostly financial [35]. In Polish hospitals, the duration of nursing duty varies; however, the 12-hour system is commonly adopted. In the hospitals where the study was conducted, nursing staff worked 12-hour shifts. There are data showing that the risk of errors in the last 4 h of on-call duty is higher than for on-call of 8 h [35]. Hobbs &Wightman [36] indicate that the risk of errors increases when nurses work longer than 12 h, regardless of whether the shifts are pre-planned, unscheduled, or voluntary [36]. Other studies show that fatigue-related accidents can increase when work is provided for four consecutive 12-hour shifts in a row. The same study found that nurses working 12-hour shifts inadvertently fell asleep during a shift when they had to work five 12-hour shifts in a row [37, 38]. In addition, Wilson et al. [39] showed that performing another job is associated with increased fatigue, lack of vigilance, irritability, and difficulties in communicating with the team. Studies by Kelly et al. also showed that additional on-call duty may adversely affect the overall health condition and the level of exhaustion of nurses [40], which may potentially pose a risk of an increase in the mortality rate of patients treated in the hospital.

These studies have discovered a positive correlation between communication disorders leading to delays in the provision of care and 30-day patient mortality. Such disorders might occur during the transfer of information, especially at the end of a long shift. The research also showed an inverse correlation coefficient between the working conditions index (WC, *r* = -0.157) and 30-day mortality. According to many researchers [4, 5, 9, 41], there is a relationship between the safety climate and patient mortality. This might be due to the fact that, in hospitals with a safety climate disorder, the potential risk of adverse events, which affect preventable mortality, is higher.

The authors recognize the inevitability of patient deaths resulting from health conditions, which are also correlated with patients’ age. Conversely, the primary focus of this study is to identify factors within the hospital work environment during the patient’s stay that may influence the risk of mortality even after hospitalization or discharge, specifically within the 30 days following completion.

### Research limitations

A significant limitation of the conducted research was the fact that data on nursing personnel as well as 30-day patient survival results were aggregated at the hospital level, without the possibility of reference to individual nurses. Nevertheless, it would be wrong to adopt the hypothesis that the work environment in any particular examined internal medicine or surgical ward differs drastically from the work environment in the entire hospital institution. Additionally, the study is constrained by the number of surveyed nurses, with 25% of respondents having their results excluded due to substantial gaps in answers. The focus on specific workplaces, namely internal medicine and surgery departments, represents another limitation. To address this, future studies are planned to broaden their scope, encompassing staff from all types of hospital departments. Furthermore, upcoming investigations will seek to expand the participant scope to include not only nurses but also doctors, paramedics, and medical caregivers.

## Conclusions

Factors related to care during hospital stay such as the organization of care at the ward level, analysis of care errors, the number of staff providing direct patient care, informing nurses about mistakes without punishment, and the possibility of nurses to challenge the decisions or actions of superiors, which concerns care providing, affect the 30-day mortality of patients after the end of hospitalization in Polish hospitals. The lower mortality rates were significantly influenced by the correct management method, i.e., the ward nurse was assessed by the staff as a good manager and, in addition, a lower rate occurred in hospitals where the nurses were informed about changes introduced based on reports on committed omissions and errors.

## Data Availability

The data analyzed in this study is subject to restrictions: the data used were explicitly made available for this study by the Ministry of Health. Any data sharing requests should be directed to the Ministry of Health. Requests to access these datasets should be directed to dep-zp@mz.gov.pl.
